# Antineoplastic Effects of Honokiol on Melanoma

**DOI:** 10.1155/2017/5496398

**Published:** 2017-01-17

**Authors:** Ruth Guillermo-Lagae, Sreevidya Santha, Milton Thomas, Emily Zoelle, Jonathan Stevens, Radhey S. Kaushik, Chandradhar Dwivedi

**Affiliations:** ^1^Department of Pharmaceutical Sciences, South Dakota State University, Brookings, SD 57007, USA; ^2^Department of Pharmacology & Therapeutics, Roswell Park Cancer Institute (RPCI), Buffalo, NY 14263, USA; ^3^Department of Biology and Microbiology; and Department of Veterinary and Biomedical Sciences, South Dakota State University, Brookings, SD 57007, USA; ^4^University of Maryland Baltimore Washington Medical Center, Glen Burnie, MD 21061, USA

## Abstract

Honokiol, a plant lignan has been shown to have antineoplastic effects against nonmelanoma skin cancer developments in mice. In this study, antineoplastic effects of honokiol were investigated in malignant melanoma models. In vitro effects of honokiol treatment on SKMEL-2 and UACC-62 melanoma cells were evaluated by measuring the cell viability, proliferation, apoptosis, cell cycle analysis, and expressions of various proteins associated with cell cycle progression and apoptosis. For the in vivo study, male nude mice inoculated with SKMEL-2 or UACC-62 cells received injections of sesame oil or honokiol for two to seven weeks. In vitro honokiol treatment caused significant decrease in cell viability, proliferation, cell cycle arrest, increased apoptosis, and modulation of apoptotic and cell cycle regulatory proteins. Honokiol caused an accumulation of cells in the G2/M phase of the cell cycle in SKMEL-2 and G0/G1 phase in UACC-62 cells. An elevated level of caspases and PARP were observed in both cell lines treated with honokiol. A decrease in the expression of various cell cycle regulatory proteins was also observed in honokiol treated cells. Honokiol caused a significant reduction of tumor growth in SKMEL-2 and UACC-62 melanoma xenografts. These findings suggest that honokiol is a good candidate for further studies as a possible treatment for malignant melanoma.

## 1. Introduction

According to the American Cancer Society, melanoma will cause 76,380 new cases and 10,130 deaths in 2016 (Cancer Facts & Figures 2016. Atlanta: American Cancer Society). Recently, much attention has been given to phytochemicals. They are being investigated for the prevention and treatment of cancer. One of those phytochemicals is honokiol (C_18_H_18_O_2_, MW 266.33), which is a naturally occurring biphenol isolated from the bark and seed cones of* Magnolia officinalis* [1, 2]. Studies have demonstrated multiple pharmacological properties of honokiol such as antioxidant [3], anti-inflammatory [4], and central nervous system depressant effects [5, 6].

Recent in vitro and in vivo studies demonstrated multiple anticancer activities of honokiol through its effect on a variety of biological pathways. Previous studies from our laboratory as well as others have showed chemopreventive effects of honokiol on UVB-induced skin cancer development in mice [7, 8]. In an earlier report, honokiol delayed the formation of papillomas in a chemically induced skin cancer protocol in mice [9]. Honokiol has anticancer effects against melanoma [10], pancreatic cancer [11], breast cancer [12], head and neck squamous cell carcinoma [13], prostate cancer, colon cancer, multiple myeloma [14–16], and squamous cell skin cancer [17]. Honokiol also potentiated apoptosis and inhibited tumor invasion through modulation of nuclear factor kappa B (NF-*κ*B) [18], inhibited angiogenesis and tumor growth [2], and inhibited tissue necrotic factor expression [18, 19].

The effects and mechanisms of action of honokiol on melanoma have not been fully explored and there are no in vivo studies on melanoma yet. In order to investigate the effects of honokiol on melanoma, in vitro effects of honokiol on cell viability, cell proliferation, and apoptosis were investigated using SKMEL-2 and UACC-62 melanoma cell lines. Animal studies were also performed by inducing xenograft tumors in nude mice and treating the animals with honokiol or vehicle. The results from these studies indicated that honokiol can be a new antineoplastic agent against melanoma.

## 2. Materials and Methods

Honokiol (98% purity) was purchased from Nacalai Tesque (Kyoto, Japan). Thiazolyl blue tetrazolium bromide (MTT) and other chemicals of analytical grade were purchased from Sigma Chemical Co. (St. Louis, MO). Leupeptin, pepstatin, and cell proliferation ELISA kit were purchased from Roche Diagnostics GmbH (Mannheim, Germany). Primary antibodies for procaspase 3, caspase 8, caspase 9, CDK6, p53, and cleaved PARP were purchased from Cell Signaling Technology (Beverly, MA). Primary antibodies for cyclin D1, cyclin D2, cyclin E, CDK-2, CDK-4, cyclin A, Cdc2p34, PCNA, caspase 3, anti-mouse IgG horseradish peroxidase-linked and anti-rabbit IgG horseradish peroxidase-linked secondary antibodies, and nitrocellulose membranes were purchased from Santa Cruz Biotechnology (Santa Cruz, CA). Anti-Kip1/p27 antibody was purchased from BD-Pharmingen (San Diego, CA), cyclin B1 and anti-Cip1/p21 antibody from Upstate Biotechnology (Lake Placid, NY).

### 2.1. Animals

Five- to six-week-old male nude mice NU/NU (NU-FOXN1(NU)) were purchased from Charles River Laboratories (Wilmington, MA, USA). Mice were housed in a climate-controlled, pathogen-free environment in the South Dakota State University's Animal Facility Building (ARW), with 12-hour light and dark cycle. They were fed an autoclaved rodent diet and water ad libitum. Nude mice's treatment preparations were sterilized by filtration with 0.22 *μ*m polyethersulfone (PES) filters from Millipore Corporation (Billerica, MA). Approval from Animal Care and Use Committee (IACUC) of South Dakota State University was obtained for all experimental protocols.

### 2.2. Melanoma Xenograft Experiments

Eighty athymic male nude mice were subcutaneously injected in the right flank with 5 × 10^6^ SKMEL-2 or UACC-62 cells and randomized into control and treatment having twenty mice in each group. Treatment groups received 50 mg/kg honokiol dissolved in sesame oil intraperitoneally (ip). Control group received equal volume of sesame oil (Loriva (R) Extra Virgin Sesame Cold Pressed Oil, Mexico), ip. The animals were treated in mornings three times per week for 2–7 weeks. This honokiol dose has been reported by other groups as attainable and nontoxic [2, 13, 16].

Over the course of the experiments, tumor volume and the mice weights were recorded every three days. External signs of toxicity were closely monitored. Vernier caliper was used to determine the length, width, and height of the tumor mass. Tumor volumes were determined by using the formula

(1) $$\begin{array}{c} V=\frac{1}{2}*\frac{4\pi }{3}*\frac{L1}{2}*\frac{L2}{2}*h, \end{array}$$

where *L*1 is the shorter diameter, *L*2 is the longer diameter, and *h* is the height [20, 21].

Animals were withdrawn from the study and euthanized when the tumors became disabling or the animal had signs of pain and discomfort.

### 2.3. Cell Lines and Culture Conditions

SKMEL-2 cells were obtained from the National Cancer Institute; UACC-62 cells were purchased from American Type Culture Collection (ATCC, Manassas, VA). Both cell lines were cultured in RPMI supplemented with 10% heat-inactivated fetal bovine serum, 100 unit/mL of penicillin, and 100 *μ*g/mL of streptomycin in a humidified atmosphere containing 5% CO_2_ at 37°C.

### 2.4. Honokiol

Honokiol was dissolved in DMSO to make a 50 mM stock solution and was diluted again in RPMI medium at different concentrations and then used immediately. The final concentration of DMSO in RPMI was 0.4% in all in vitro assays.

### 2.5. Cell Viability Assay

MTT assay as routinely used in our laboratory [17] was employed to determine cell viability. SKMEL-2 or UACC-62 cells were plated at 10,000 cells per well into 96-well plates. Cells were treated with control media, 10 *μ*M, 25 *μ*M, 50 *μ*M, 75 *μ*M, or 100 *μ*M of honokiol and incubated for 12, 24, 48, or 72 hours to observe the effects on cell viability. Each experiment was repeated four times.

### 2.6. Cell Proliferation Assay

The bromodeoxyuridine incorporation (BrdU) assay was performed [17] to determine cell proliferation by using the ELISA kit (Roche Diagnostics, GmbH, Manheim, Germany). SKMEL-2 or UACC-62 cells were plated at 10,000 cells per well in 96-well plates. After 24 hours, the cells were treated with control media, 10 *μ*M, 25 *μ*M, 50 *μ*M, 75 *μ*M, or 100 *μ*M of honokiol for 12, 24, and 48 hours. The absorbance was measured at 450 nm and 650 nm in a Spectra Max M2 microplate reader (Molecular Devices, Sunnyvale, CA, USA). Each experiment was repeated three times.

### 2.7. TUNEL: DNA Fragmentation Apoptosis Assay

The DNA fragmentation in SKMEL-2 and UACC-62 cells was quantified using an Apo-BrdU TUNEL assay kit (Molecular Probes, Eugene, OR, USA) per the manufacturer's protocol as reported earlier [17]. Cells were treated with control media, 50 *μ*M, 75 *μ*M, and 100 *μ*M of honokiol for 12, 24, 48, or 72 hours and harvested and fixed shortly after the completion of treatment. DNA nicks in fixed cells were labeled with BrdU; then samples were incubated with Alexa Fluor 488-labeled anti-BrdU antibody. The cells were analyzed using flow cytometry. Both positive and negative controls were run with each assay. The experiment was repeated four times.

### 2.8. Immunoblot

Western blotting was used to determine the levels of protein expression in SKMEL-2 and UACC-62 cells treated with varying concentrations of honokiol. SKMEL-2 or UACC-62 cells (1.5 × 10^6^) were plated in 100 mm culture dishes. The cells were treated with honokiol 0 *μ*M, 25 *μ*M, 50 *μ*M, 75 *μ*M, or 100 *μ*M for 12 and 24 hours. After each treatment, cells were lysed and protein concentrations were determined using BCA protein assay kit (Pierce, Rockford, FL, USA). Equal amounts of proteins were denatured and separated using sodium dodecyl sulfate-polyacrylamide gel electrophoresis and transferred onto nitrocellulose membranes. The membranes were blocked in 5% nonfat milk and incubated with the appropriate primary antibodies followed by secondary antibody. The proteins were detected using the ECL Plus Detection System (Amersham Biosciences, Piscataway, NJ). The band densities were quantified using the UVP Biochem Gel Documentation System (UVP Inc., Upland, CA, USA). Consistent protein loading was ensured by probing each membrane for *β*-actin. The Western blots were repeated 3–5 times. A representative blot is reported.

### 2.9. Cell Cycle DNA Analysis

Subconfluent SKMEL-2 or UACC-62 cells were treated with control media or honokiol 25 *μ*M, 50 *μ*M, 75 *μ*M, and 100 *μ*M for 12, 24, and 48 hours. Following treatment, the cells were fixed and then treated with RNase A. After this, propidium iodide was added. The samples were analyzed using BD FACScan™ flow cytometer and Cell Quest Software (BD Biosciences, San Joes, CA, USA) [17]. The experiment was repeated four times.

### 2.10. Statistical Analysis

ANOVA followed by Tukey posttest was applied to compare the statistical difference between the honokiol treatment group and control group in the in vitro experiments. For the in vivo experiments Mann–Whitney* U* test was used. Significance in all the experiment was considered to be *P* < 0.05. Values were expressed as the mean ± the standard error of the mean. Xenograft and in vitro experiments' data were analyzed using INSTAT software Graph Pad (San Diego, CA).

## 3. Results

### 3.1. Honokiol Treatment Decreased Cell Viability in SKMEL-2 and UACC-62 Cells

**Figure 1:**
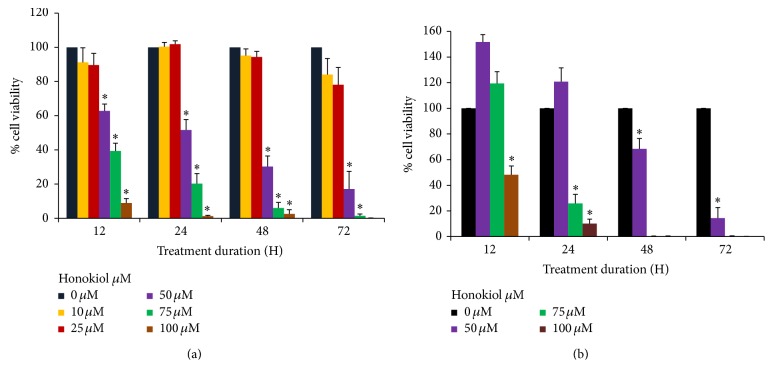
Honokiol decreased cell viability in SKMEL-2 (a) and UACC-62 (b) cells as evaluated by MTT assay. Cells were treated with honokiol 0–100 *μ*M for 12, 24, 48, and 72 hours. At the end of respective treatments, MTT assay was performed in each cell line. ^*∗*^*P* < 0.05 indicates statistically significant decrease in honokiol treated groups as compared with the control. *N* = 4.

Both SKMEL-2 and UACC-62 cells were treated with DMSO or varying concentrations (0–100 *μ*M) of honokiol for 12, 24, 48, and 72 h and cell viability was determined by MTT assay. Effect of honokiol on SKMEL-2 and UACC-62 cells viability is presented in Figures 1(a) and 1(b), respectively. Honokiol treatment resulted in a decrease in cell viability in both SKMEL-2 and UACC-62 cells. Significant reduction in cell viability was observed at 50 *μ*M concentration and above in both cell lines. In SK-MEL-2 cells 24 h treatment with 50–100 *μ*M of honokiol showed significant decrease of 57%–98% in cell viability compared to the control. In UACC-62 cells, 24-hour treatment with 75−100 *μ*M honokiol showed significant decrease (^*∗*^*P* < 0.05) in cell viability of 74.2% and 89.9%, respectively.

### 3.2. Honokiol Treatment Decreased Cell Proliferation in SKMEL-2 and UACC-62 Cells

**Figure 2:**
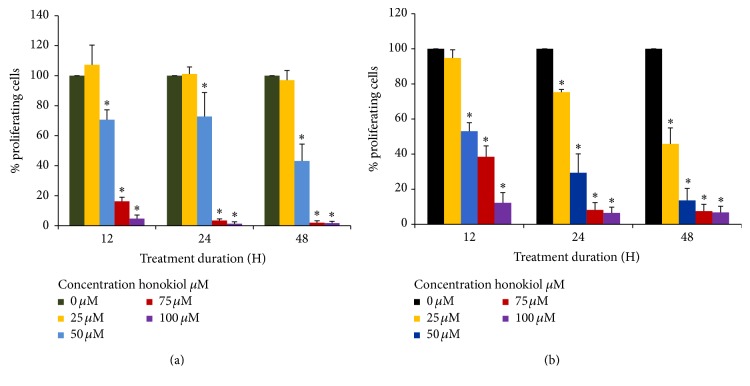
Effects of honokiol on cell proliferation in SKMEL-2 (a) and UACC-62 (b) cells. Cells were treated with 0–100 *μ*M honokiol for 12, 24, and 48 hours. After the respective treatments, BrdU assay was performed as discussed in Section 2. ^*∗*^*P* < 0.05 indicates statistically significant decrease in honokiol treated groups as compared with the control. *N* = 3.

BrdU cell proliferation ELISA was conducted to determine the cell proliferation rate after treatment with 0–100 *μ*M/L of honokiol for 12, 24, and 48 hrs. The results of the BrdU assay for cell proliferation of SK-MEL-2 and UACC-62 cells are shown in Figures 2(a) and 2(b), respectively. After 12 hours, 50, 75, and 100 *μ*M concentrations of honokiol significantly decreased SK-MEL-2 cell proliferation by 25%, 82%, and 93% (^*∗*^*P* < 0.05), respectively. Honokiol treatments of 75 *μ*M and 100 *μ*M resulted in almost complete inhibition of cell proliferation after 24 h treatment. In UACC-62 cells, honokiol at 50–100 *μ*M for 12 hours reduced cell proliferation by 47.0% to 87.3% (^*∗*^*P* < 0.05). After 48-hour treatment, 25–100 *μ*M honokiol resulted in an inhibition of cell proliferation by 54.2% to 93.2% (^*∗*^*P* < 0.05) as compared to the control.

### 3.3. Honokiol Induces Apoptotic Death in SKMEL-2 and UACC-62 Melanoma Cells

**Figure 3:**
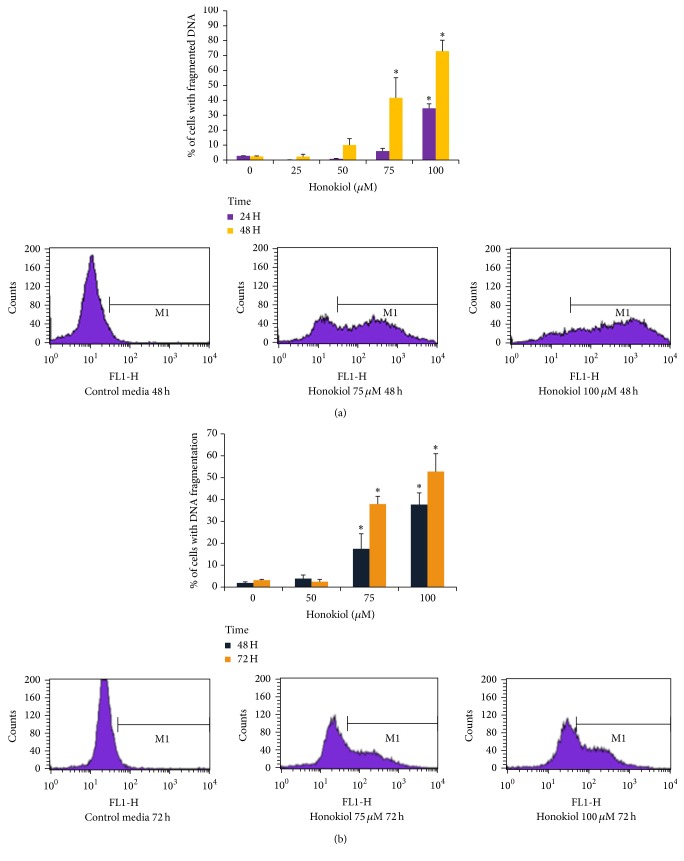
Effects of honokiol on DNA fragmentation by TUNEL assay and flow cytometry in SKMEL-2 (a) and UACC-62 (b) cells. Cells were treated with 0–100 *μ*M honokiol for 24, 48, or 72 hours. After the respective treatments, TUNEL assay was performed by using APO-BrdU TUNEL assay kit. The extent of DNA fragmentation was quantified by computational analysis of cells staining positive for BrdU using CellQuest software. The bars indicates the percentages of apoptotic cells with fragmented DNA. ^*∗*^*P* < 0.05 indicates statistical significance in honokiol treated groups as compared to the control cells. *N* = 4.

TUNEL assay was performed to investigate the effects of honokiol on DNA fragmentation, which is a hallmark of the end stages of apoptosis. SK-MEL-2 and UACC-62 cells were treated with 0−100 *μ*M of honokiol for 24–72 hours. In SK-MEL-2 cells, 48 h treatment with 50, 75, and 100 *μ*M of honokiol induced 10, 40, and 75% DNA fragmentation, respectively (Figure 3(a)). In UACC-62 cells, 48 h treatment with 75 and 100 *μ*M honokiol induced 17.5% and 37.7% DNA fragmentation, respectively. Treatment for 72 hours with 75 and 100 *μ*M honokiol increased the DNA fragmented cells to 37.9% and 52.7%, respectively (^*∗*^*P* < 0.05) (Figure 3(b)).

### 3.4. Honokiol Induces G_0_/G_1_ Phase Cell Cycle Arrest in UACC-62 and G_2_/M Phase Cell Cycle Arrest in SKMEL-2 Cells

**Figure 4:**
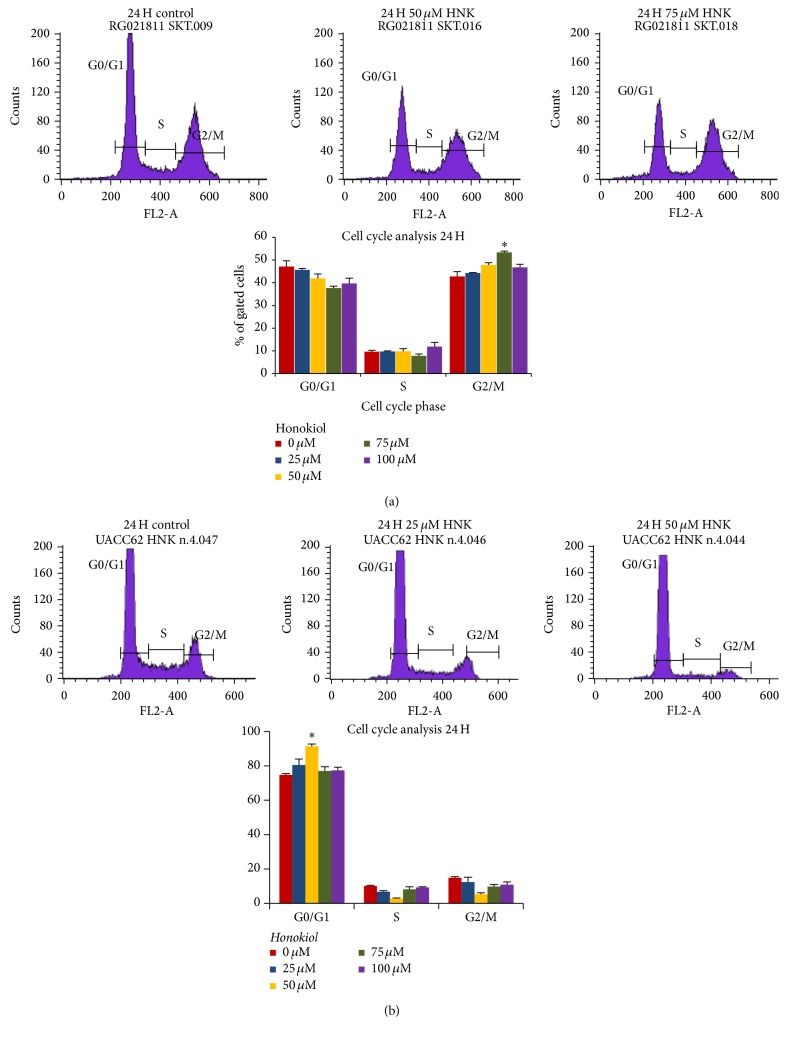
Effects of honokiol on the distribution of cells in different phases of the cell cycle. Cells were treated with 0–75 *μ*M honokiol for 12, 24, and 48 hours and stained with propidium iodide. Distribution of cells in different phases of cell cycle were analyzed by flow cytometer. The percentage of cells in each cell cycle phases after 24 h treatment in SKMEL-2 and UACC-62 cells is shown in (a) and (b), respectively.

We analyzed the effects of honokiol treatment on cell cycle phase distribution in SKMEL-2 and UACC-62 cells. Both cells were plated at 0.4 × 10^6^ cells/well in six-well plates and were treated with 25 *μ*M–100 *μ*M of honokiol or media for 12–48 hours. As shown in Figure 4(a), 75 *μ*M honokiol treatment for 24 hours in SK-MEL-2 cells showed a significant accumulation of cells in the G2/M phase (54%) as compared to the control (42%). After 24 h, UACC-62 cells treated with 50 *μ*M honokiol showed a significant difference (*P* < 0.05) in the amount of cells in G0/G1 phase (92.6%) as compared with the control (73.9%) (Figure 4(b)). Representative DNA histograms of SK-MEL-2 and UACC-62 cells are shown in Figure 4.

### 3.5. Honokiol Modulates Proteins Involved in Apoptosis and Cell Cycle Control

**Figure 5:**
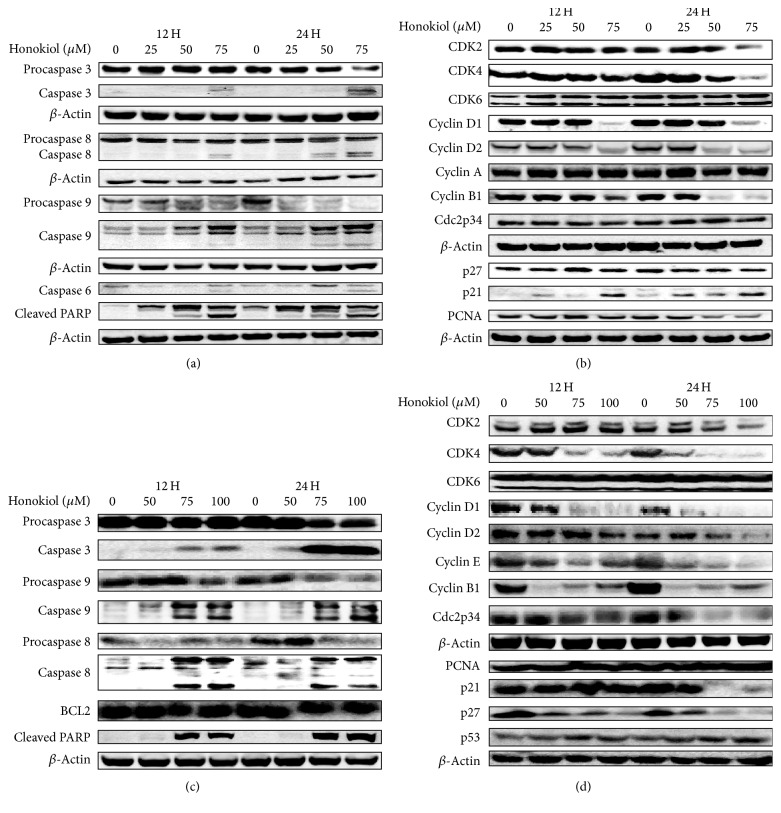
Effects of honokiol on proteins associated with apoptosis and cell cycle in SKMEL-2 (a, b) and UACC-62 (c, d) cells. Cells were treated with 0–75 *μ*M honokiol for 12 and 24 hours. Following treatment, total cell lysates were prepared and equal amounts of proteins were separated by SDS-PAGE and subjected to western immunoblotting. Honokiol induces apoptosis in both cells as observed by the activation of different caspases and PARP cleavage. *β*-Actin was used to verify equal loading of the samples.

Cells were treated with 25–75 *μ*M honokiol for 12 to 24 hrs and analyzed for the expression of proteins involved in apoptosis and cell cycle regulation. Honokiol modulated various proteins in SKMEL-2 cell line in a concentration and time dependent fashion. Effect of honokiol on various protein expressions in SKMEL-2 cells is shown in Figures 5(a) and 5(b). It increased the activation of proapoptotic proteins, cleaved caspases 3, 6, 8, 9, and cleaved PARP while it decreased procaspases 3 and 9 levels (Figure 5(a)). In the same cell line, honokiol decreased the expression of cell cycle regulatory proteins CDK2, CDK4, cyclin D1, cyclin D2, cyclin B1, and PCNA (Figure 5(b)). The level of CDK inhibitor p21 was increased with honokiol treatment, while p27, CDK6, cyclin A, and Cdc2p34 did not change at any of the concentrations and times evaluated (Figure 5(b)).

Honokiol's effects in the UACC-62 cells included increasing caspases 3, 8, and 9 and cleaved PARP, while decreasing procaspases 3, 8, and 9 and PARP (Figure 5(c)). Honokiol also decreased the expression of cell cycle proteins CDK2, CDK4, cyclin D1, cyclin D2, cyclin E, cyclin B1, Cdc2p34, p21, and p27 (Figure 5(d)). Honokiol caused an increase in p53 levels while PCNA proteins expressions did not change with the honokiol treatments (Figure 5(d)).

### 3.6. Honokiol Significantly Decreased Tumor Growth in SKMEL-2 and UACC-62 Cells Xenografts in Nude Mice

**Figure 6:**
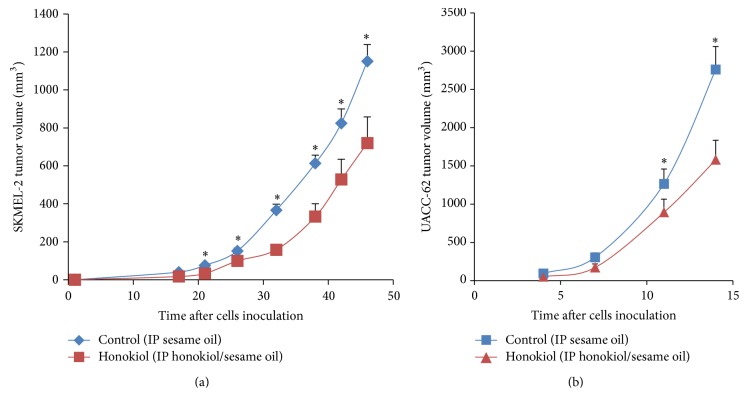
Effects of honokiol on xenograft model tumor volume. Xenograft protocol was performed as discussed in Section 2. Tumor volume was smaller in the honokiol treated animals as compared to the control group in SKMEL-2 (a) and UACC-62 (b) group (^*∗*^*P* < 0.05). *N* = 20.

Honokiol treatment did not affect animals' weights, tumor cells characteristics, or architecture (data not shown). At the end of the experiment the honokiol treated animals had smaller tumors overall than the control animals. In SKMEL-2 cells inoculated animals, a significant reduction (*P* < 0.05) of approximately 40% in tumor volume was observed (Figure 6(a)). Since tumor in UACC-62 cells inoculated animals was very aggressive, thus animals were sacrificed only 15 days after inoculation. A significant reduction of approximately 50% in tumor volume was observed with the honokiol treatment (*P* < 0.05) at the end of experiment (Figure 6(b)).

## 4. Discussion

The growth inhibitory effects of honokiol alone or in combination with other agents on melanoma cells have been previously reported [2, 10, 12]. Our studies provided evidence that honokiol is effective in suppressing the growth of melanoma cells in vitro and in vivo. Honokiol reduced the cell viability and proliferation of melanoma cell lines SKMEL-2 and UACC-62 in a concentration and time dependent fashion. Honokiol caused G2/M cell cycle arrest in SKMEL-2 and G0/G1 cell cycle arrest in UACC-62 cells. Honokiol induced DNA fragmentation and apoptosis in both cell lines.

In order to investigate the possible mechanisms of action of honokiol in melanoma cell lines, the key proteins involved in apoptosis and cell cycle regulation were evaluated by Western blots. Induction of apoptosis may involve the activation of caspases. Western blot analysis indicated that honokiol increased the activation of proapoptotic proteins and PARP cleavage, which are hallmarks of apoptosis in SKMEL-2 and UACC-62 cell lines. This study suggests the involvement of both extrinsic and intrinsic pathways of apoptosis in SKMEL-2 and UACC-62 cell lines as observed by the activation of caspase 8 and caspase 9 in honokiol treated cells [22]. In addition, we found the activation of the executioner caspases 3 and 6 in SKMEL-2 and caspase 3 in UACC-62 cells.

Proteins involved in the regulation of the cell cycle were also modulated by honokiol. The cell cycle arrest in SK-MEL-2 cells was also associated with a decrease in cyclin D1, cyclin D2, CDK2, and CDK4. However, the expressions of Cdc2p34, CDK6, and cyclin A seemed not to change at any of the concentrations and times evaluated. Also, the CDK inhibitor p21 was upregulated with honokiol treatment, while p27 did not show significant difference. Activation of cyclin-CDK complexes plays a central role in cell cycle progression and arrest processes [22, 23]. Cyclin B/Cdc2 complex regulates the G2/M transition.

It has been reported that SKMEL-2 cell line barely express p21 [24, 25]. Studies also reported that p21 inhibit cell cycle progression by inhibiting cyclin/CDK complexes and by inhibiting proliferating cell nuclear antigen (PCNA) function. Binding of p21 to PCNA in p53 deficient cells causes G2/M cell cycle arrest [26]. Given the increase in p21, decrease in PCNA, and accumulation of cells in the G2/M cell cycle phase in the SKMEL-2 cells treated with honokiol, the G2/M cell cycle arrest may have occurred due to the binding of p21 to PCNA. This hypothesis may need to be confirmed by immunoprecipitation to show the increased interaction between p21 and PCNA. Other studies also demonstrated the effects of p21 during G2/M phase [27] and correlation between p21 protein expression and progression of primary melanomas [28–30]. The transition from the G2 into the M phase requires activation of cyclin dependent kinase (CDK) by increased accumulation of the regulatory subunit of cyclin B1 to a threshold level [31]. Honokiol strongly decreased the expression of cyclin B1 in SKMEL-2 cells, explaining in part the cell cycle arrest in G2/M phase.

G0/G1 cell cycle arrest was observed with honokiol treatment in UACC-62 cells. Hence, cell lysates were analyzed for the proteins CDK2, CDK4, cyclin D1, cyclin D2, and cyclin E which are involved in G0/G1 phase [32–36]. Honokiol downregulated all these proteins in UACC-62 cells. In cell cycle, the cyclin D/Cdk4 and Cdk6 drives the cells through the early G1 phase, while cyclin E/cdk2 is a key factor in the later G1 phase and promotes transition into S phase. The modulating effect of honokiol on CDK2, CDK4, and cyclins D1, D2, and E may explain the UACC-62 cell cycle arrest at G0/G1 phase.

UACC-62 cell line has a wild type p53 protein and mutations in the p14ARF and p16 genes. Honokiol treatment caused a modest increase in the expression of p53 in UACC-62 cell line. Kichina et al. [37] have reported that overexpression of wild type 53 is capable of growth inhibition in melanoma cell lines. Thus, increase in wild type p53 protein expression observed with the honokiol treatment may be of importance in the growth inhibition observed in UACC-62 cells.

In order to study honokiol effects in in vivo systems, xenograft tumors from the melanoma cells were established in male nude mice. The tumors from UACC-62 cells were more aggressive than those of SKMEL-2 cells, although the same number of cells was injected. The difference in the aggressiveness of the tumors was seen from an extensive degree of necrosis present in the UACC-62 tumors compared to occasional multicellular necrosis in the SKMEL-2 cells tumors. This can be explained by the fact that SKMEL-2 cells form slow growing tumors. Nevertheless, a significant (*P* < 0.05) inhibition in tumor volume was observed in both groups of animals treated with honokiol. At the end of the experiment, honokiol treatment caused a significant (*P* < 0.05) reduction in the tumor volumes in the SKMEL-2 and the UACC-62 cells treated tumors.

Honokiol has multiple molecular targets as underlying mechanisms associated with the anticancer properties. It interacts with various molecular targets including nuclear factor kappa B (NF-*κ*B) pathway, PI3K/mTOR pathway, signal transducers and activator of transcription 3 (STAT3), mitogen-activated protein kinases, epidermal growth factor receptor (EGFR) signaling, and cyclooxygenase 2 (COX-2) [1]. Honokiol induces caspase-dependent apoptosis and enhances the cytotoxicity of fludarabine, cladribine, and chlorambucil in B-cell chronic lymphocytic leukemia (B-CLL) cells [38]. In multidrug-resistant (MDR) KB cells, combined treatment with honokiol and paclitaxel synergistically augment the cytotoxicity of paclitaxel by inhibiting the EGFR-STAT3 signaling pathway and downregulating multiple antiapoptotic proteins. In addition, honokiol enhances the in vivo efficacy of paclitaxel in KB-8-5 xenograft tumors [39].

Honokiol's effects have been studied in other cancer xenograft models such as overexpressing VEGF-D Lewis lung carcinoma cells [40]; transformed endothelial cell line SVR [2]; Ovarian carcinoma SKOV3 cells [41]; colorectal cancer [16]; head and neck cancer cell lines Cal-33 and 1483 [23]; malignant bone tumor human chondrosarcoma cells [42]; mouse 4T1 breast cancer cells [43]; lung cancer A549 cells [44]; and hepatocellular carcinoma [45]. Honokiol showed in all studies a reduction in tumor progression alone or in combination with chemotherapy drugs. Overall, the evidence presented in our studies indicated that honokiol is a good candidate for future studies as an antineoplastic agent for the treatment of melanoma.
